# Supplementary figures and images for: Structure and Antigenicity of the Porcine Astrovirus 4 Capsid Spike
Source: Viruses. 2024 Oct 11;16(10):1596. doi: 10.3390/v16101596 (PMC11512355; doi:10.3390/v16101596)

250 kD  
150  
100  
75  
50  
37  
25  
20  
15  
10

X X X X

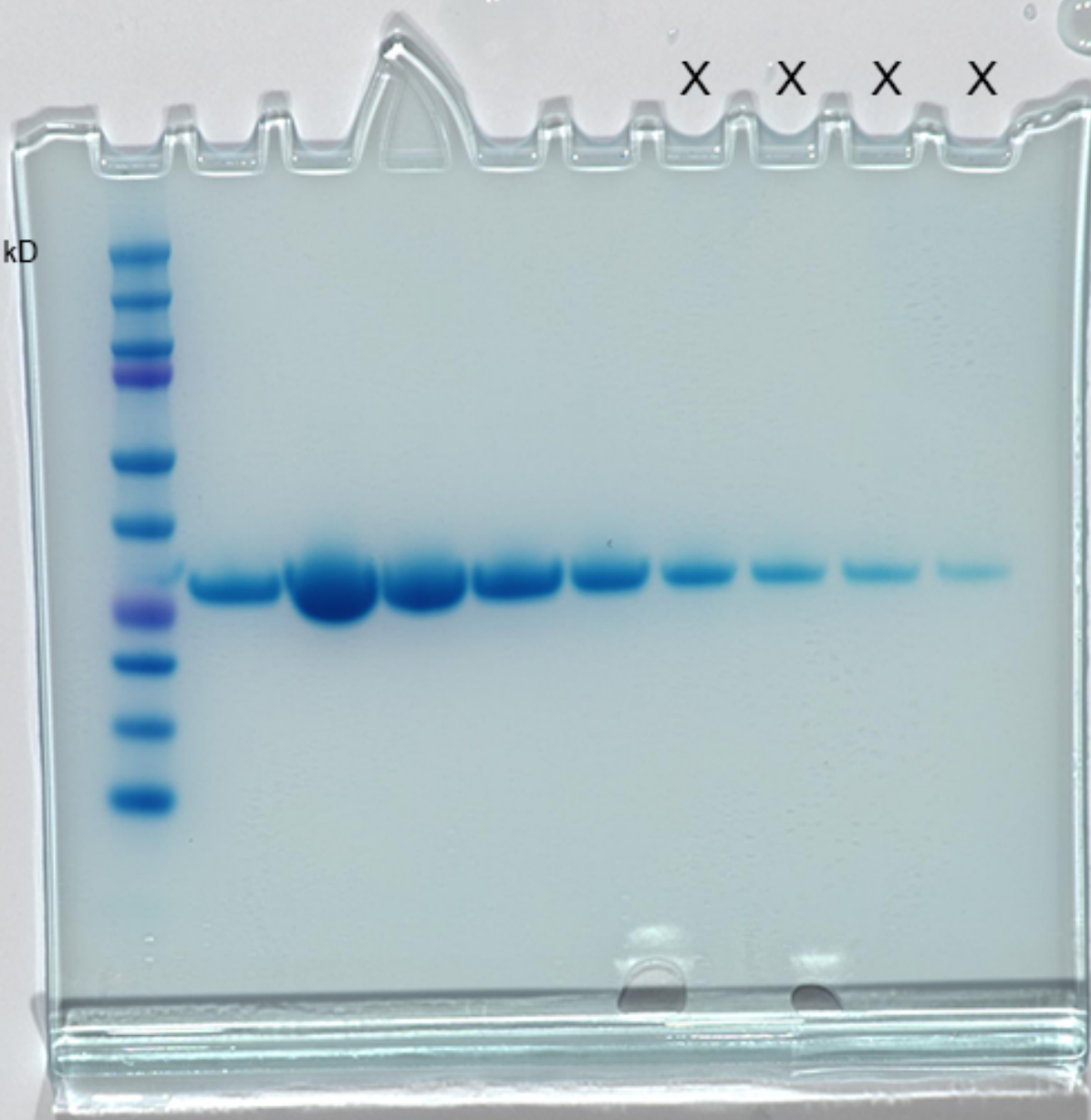

Supplement: Supplementary file 1 [file viruses-16-01596-s001.zip › viruses-3239320-supplementary.pdf]
